# Supplementary material for: Cytotoxic Metabolites from Calophyllum tacamahaca Willd.: Isolation and Detection through Feature-Based Molecular Networking
Source: Metabolites. 2023 Apr 23;13(5):582. doi: 10.3390/metabo13050582 (PMC10222348; doi:10.3390/metabo13050582)
Supplement: Supplementary file 1 [file metabolites-13-00582-s001.zip › metabolites-2343618-supplementary.pdf]

Supporting information

Cytotoxic metabolites from *Calophyllum tacamahaca* Willd.: isolation and detection through Feature-Based Molecular Networking

Elise Gerometta [1], Gaëtan Herbette [2], Elnur Garayev [3], Arnaud Marvilliers [1], Jean-Valère Naubron [2], Carole Di Giorgio [4], Pierre-Eric Campos [1,5], Patricia Clerc [1], Allison Ledoux [6], Michel Frederich [6], Béatrice Baghdikian [3], Isabelle Grondin [1] and Anne Gauvin-Bialecki [1,*]

[1] Laboratoire de Chimie et de Biotechnologie des Produits Naturels, Faculté des Sciences et Technologies, Université de La Réunion, St Denis, La Réunion, France; elise.gerometta@univ-reunion.fr (E.Ge.); arnaud.marvilliers@univ-reunion.fr (A.M.); patricia.clerc@univ-reunion.fr (P.C.); isabelle.grondin@univ-reunion.fr (I.G.); anne.bialecki@univ-reunion.fr (A.G-B)

[2] Aix-Marseille Univ, CNRS, Centrale Marseille, FSCM, Spectropole, Campus de St Jérôme – Service 511, Marseille, France; gaetan.herbette@univ-amu.fr (G.H.); jean-valere.naubron@univ-amu.fr (J-V.N.)

[3] Aix Marseille Univ, Avignon Université, CNRS, IRD, IMBE, Marseille, France, Faculty of Pharmacy, Service of Pharmacognosy, Marseille, France; elnur.garayev@univ-amu.fr (E.Ga.); beatrice.baghdikian@univ-amu.fr (B.B.)

[4] Aix-Marseille Univ, Avignon Université, CNRS, IRD, IMBE, Marseille, France, Faculty of Pharmacy, Service of environmental mutagenesis, Marseille, France; carole.di-giorgio@univ-amu.fr (C.D.)

[5] Institut de Chimie Organique et Analytique, Université d'Orléans – CNRS - Pôle de chimie, rue de Chartres – UMR 6759, BP6759, 45067 Orléans Cedex 2, France; pierre-eric.campos@univ-orleans.fr (P-E.C.)

[6] Université de Liège, Département de Pharmacie, Centre Interfacultaire de Recherche sur le Médicament (CIRM), Laboratoire de Pharmacognosie, Campus du Sart-Tilman, Quartier Hôpital, Liège, Belgium; allison.ledoux@uliege.be (A.L.); m.frederich@uliege.be (M.F.)

* Correspondence: anne.bialecki@univ-reunion.fr

Supporting information

Figure S1. HRESIMS spectrum for isocaloteysmannic acid (1). 3
Figure S2. ^{1}H NMR (600 MHz, CD_3OD) spectrum for isocaloteysmannic acid (1). 3
Figure S3. ^{13}C NMR (150 MHz, CD_3OD) spectrum for isocaloteysmannic acid (1). 4
Figure S4. ^{1}H-^{1}H COSY NMR (600 MHz, CD_3OD) spectrum for isocaloteysmannic acid (1). 4
Figure S5. ^{1}H-^{13}C HSQC NMR (600 MHz, CD_3OD) spectrum for isocaloteysmannic acid (1). 5
Figure S6. ^{1}H-^{13}C HMBC NMR (600 MHz, CD_3OD) spectrum for isocaloteysmannic acid (1). 5
Figure S7. Plot of Boltzmann-weighted calculated NMR δ ^{13}C of 4R,10R,11R and 4S,10R,11R isomers versus experimental NMR δ ^{13}C of isocaloteysmanic acid (1). Statistics for the regression of calculated versus experimental chemical shifts for both isomers. The slope, the intercept, the correlation coefficient (r) are followed by their standard error on the last digit(s) in brackets. F is the Fisher F-statistic and s the standard error of the fit. 11
Figure S8. UV (left) and ECD (right) spectra calculated using SMD(CH_3OH)/CAM-B3LYP/6-31++G(d,p)//GD3BJ-B3LYP/6-311G(d,p) level for (4R,10R,11R) (red) and (4S,10R,11R) (blue). 12
Figure S9. Conformations A_1 to A_6 selected to build the UV and ECD spectra of the diastereomer (4R,10R,11R). Geometries optimized to the level GD3BJ-B3LYP/6-311G(d,p). 13
Figure S10. Conformations B_1 to B_6 selected to build the UV and ECD spectra of the diastereomer (4S,10R,11R). Geometries optimized to the level GD3BJ-B3LYP/6-311G(d,p). 13
Figure S11. HRESIMS spectrum for 6-(4-hydroxy-3-methylbutyl)-1,5-dihydroxyxanthone (3). 14
Figure S12. ^{1}H NMR (600 MHz, CD_3OD) spectrum for 6-(4-hydroxy-3-methylbutyl)-1,5-dihydroxyxanthone (3). 14
Figure S13. ^{1}H-^{1}H COSY NMR (600 MHz, CD_3OD) spectrum for 6-(4-hydroxy-3-methylbutyl)-1,5-dihydroxyxanthone (3). 15
Figure S14. ^{1}H-^{13}C HSQC NMR (600 MHz, CD_3OD) spectrum for 6-(4-hydroxy-3-methylbutyl)-1,5-dihydroxyxanthone (3). 15
Figure S15. ^{1}H-^{13}C HMBC NMR (600 MHz, CD_3OD) spectrum for 6-(4-hydroxy-3-methylbutyl)-1,5-dihydroxyxanthone (3). 16

Table S1. Boltzmann weighted populations of conformations 1-36 used for $DP4^+$ analysis of isomer 4R,10R,11R. 6
Table S2. Boltzmann weighted populations of conformations 1-36 used for $DP4^+$ analysis of isomer 4R,10R,11S. 7
Table S3. Boltzmann weighted populations of conformations 1-36 used for $DP4^+$ analysis of isomer 4S,10R,11R. 9
Table S4. Boltzmann weighted populations of conformations 1-36 used for $DP4^+$ analysis of isomer 4S,10R,11S. 10
Table S5. Coefficients of determination R^2 of the linear regressions made between experimental chemical shifts and theorical chemical shifts of each isomer. 11
Table S6. Enthalpies and Boltzmann populations of conformations A_1-A_{14} of (4R,10R,11R) and conformations B_1-B_{14} of (4S,10R,11R), calculated using GD3BJ-B3LYP/6-311G(d,p) level. 12

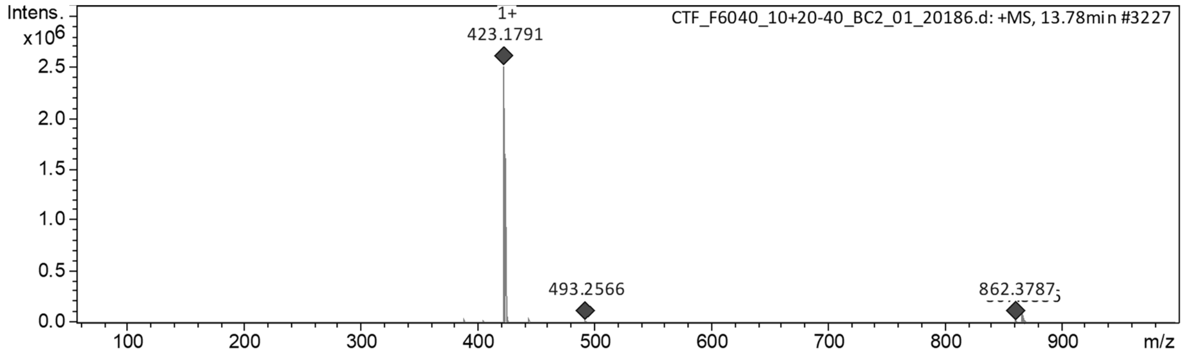

Figure S1. HRESIMS spectrum for isocaloteysmannic acid (1).

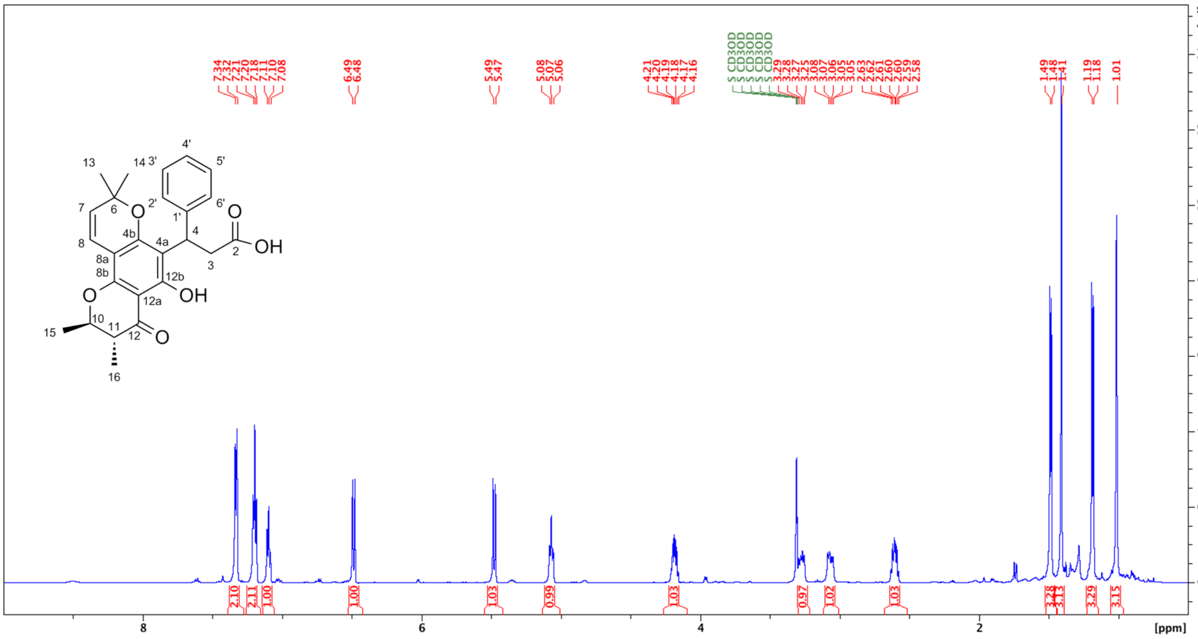

Figure S2. ^{1}H NMR (600 MHz, CD_3OD) spectrum for isocaloteysmannic acid (1).

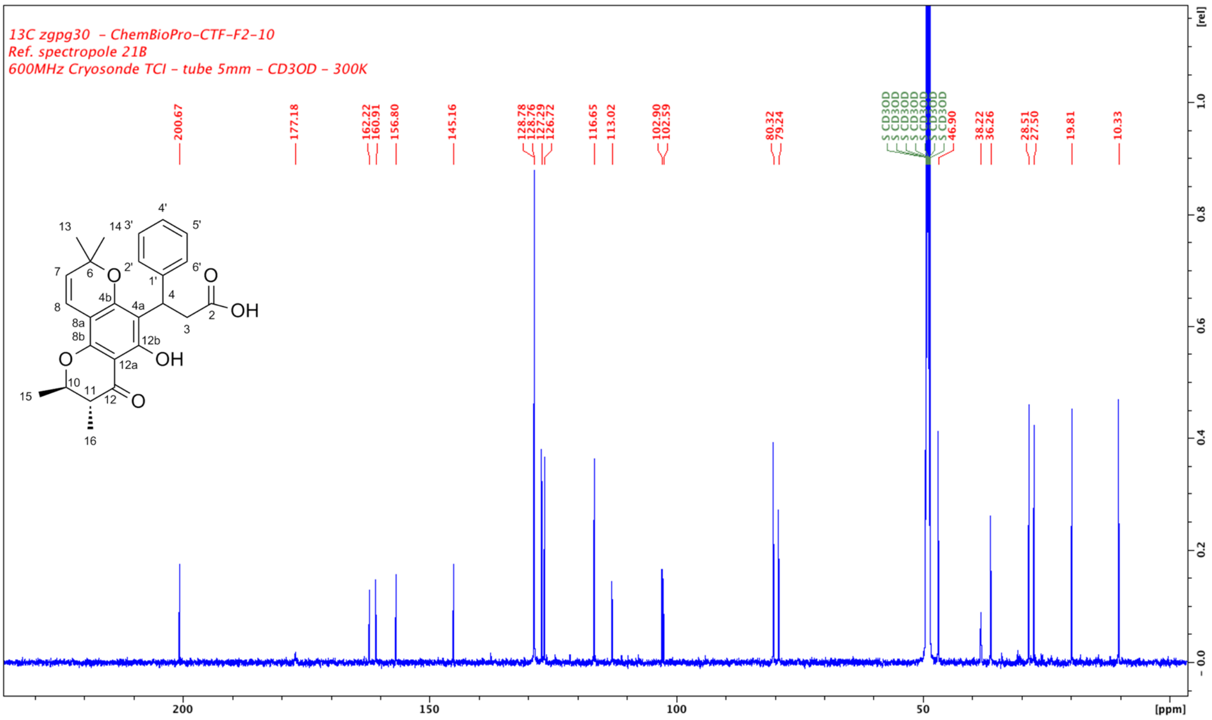

Figure S3. ^{13}C NMR (150 MHz, CD_3OD) spectrum for isocaloteysmannic acid (1).

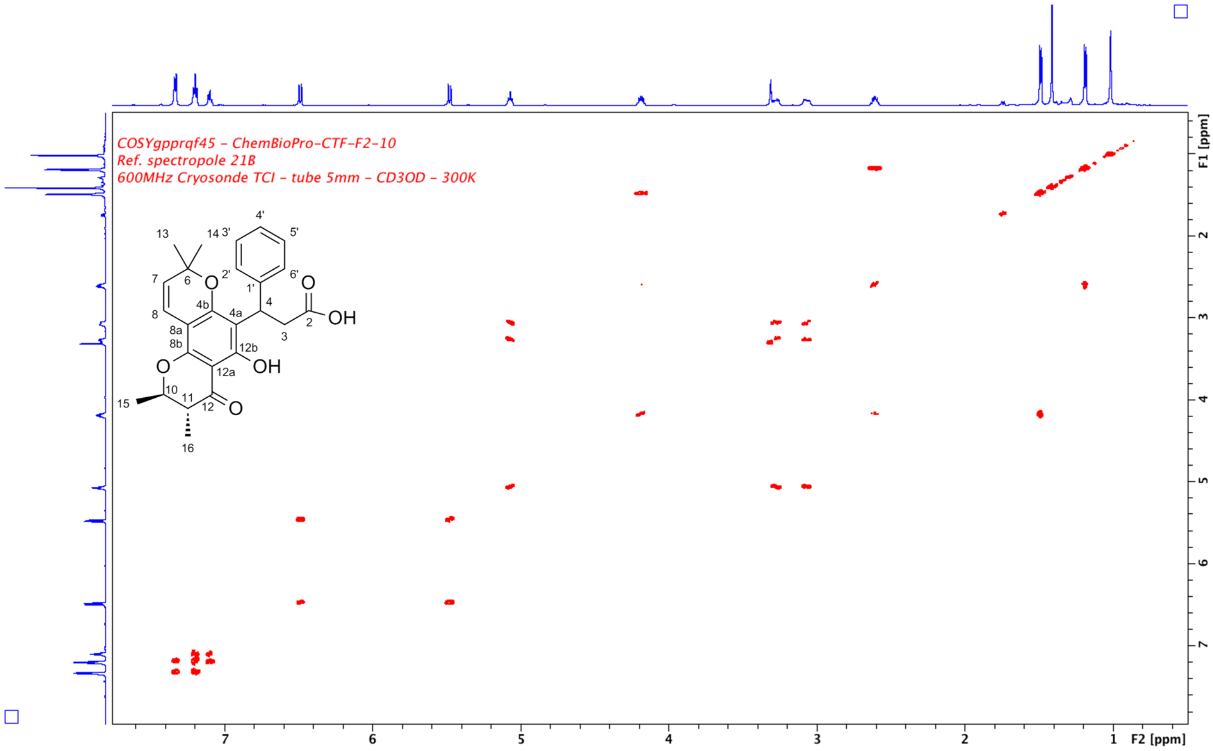

Figure S4. ^1H-^1H COSY NMR (600 MHz, CD_3OD) spectrum for isocaloteysmannic acid (1).

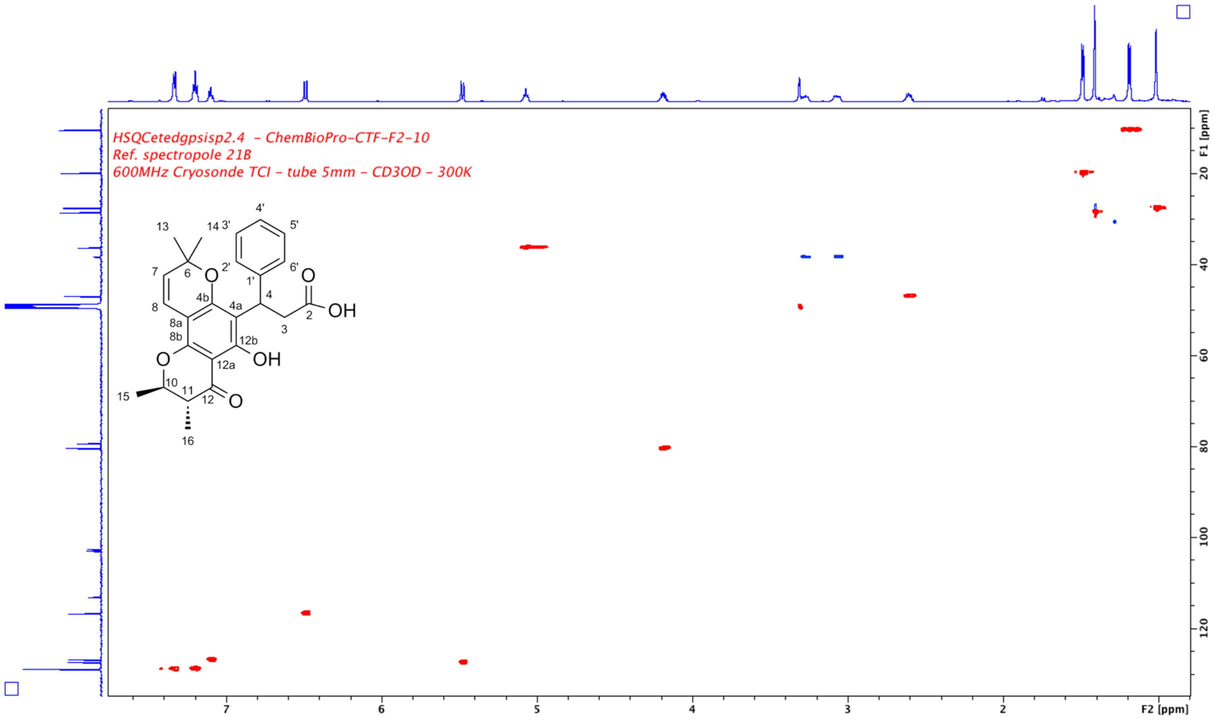

Figure S5. ^{1}H-^{13}C HSQC NMR (600 MHz, CD_3OD) spectrum for isocaloteysmannic acid (1).

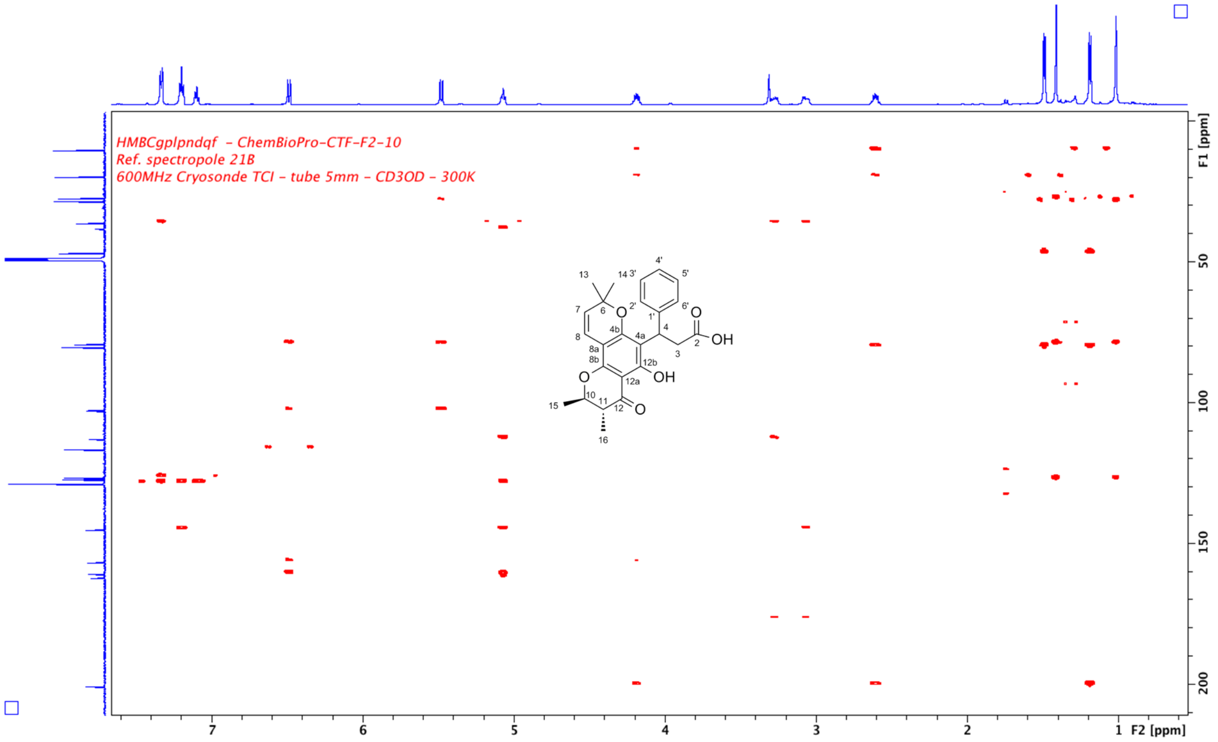

Figure S6. ^{1}H-^{13}C HMBC NMR (600 MHz, CD_3OD) spectrum for isocaloteysmannic acid (1).

Supporting information

Table S1. Boltzmann weighted populations of conformations 1-36 used for DP4⁺ analysis of isomer 4*R*,10*R*,11*R*.

**File name**	**Dipole**	**Energy**	**Ranking delta energy in a.u.**	**Rank**	**Ranking delta energy in kJ/mol**	**Ni/N0**	**Ni**	**Boltzmann population in %**
s-smd-B3LYP_6-31pgss_trans_B_B_ax_ax_01.log	9.3317	-1419.820508	0.00424343	30	11.14	0.011172796	0.001510071	0.15
σ–σμδ–Β3ΛΨΠ_6–31πγσσ_τρανσ_Β_Β_αξ_αξ_02.λογ	6.2038	-1419.821567	0.00318451	24	8.36	0.034294953	0.004635169	0.46
σ–σμδ–Β3ΛΨΠ_6–31πγσσ_τρανσ_Β_Β_αξ_αξ_21.λογ	5.089	-1419.821833	0.00291806	23	7.66	0.045476647	0.006146442	0.61
s-smd-B3LYP_6-31pgss_trans_B_B_ax_ax_22.log	10.241	-1419.820444	0.0043077	31	11.31	0.010437579	0.001410702	0.14
s-smd-B3LYP_6-31pgss_trans_B_B_ax_ax_24.log	8.0931	-1419.820856	0.00389534	28	10.23	0.016153692	0.002183269	0.22
s-smd-B3LYP_6-31pgss_trans_B_H_ax_ax_01.log	9.1836	-1419.822249	0.0025027	22	6.57	0.070605798	0.009542798	0.95
s-smd-B3LYP_6-31pgss_trans_B_H_ax_ax_02.log	6.0979	-1419.822262	0.00248946	21	6.54	0.071602854	0.009677556	0.97
s-smd-B3LYP_6-31pgss_trans_B_H_ax_ax_21.log	4.6583	-1419.822279	0.0024721	20	6.49	0.072931536	0.009857136	0.99
s-smd-B3LYP_6-31pgss_trans_B_H_ax_ax_22.log	8.934	-1419.821	0.00375115	27	9.85	0.01881891	0.002543489	0.25
s-smd-B3LYP_6-31pgss_trans_B_H_ax_ax_24.log	7.6215	-1419.821368	0.00338343	25	8.88	0.027780084	0.003754645	0.38
s-smd-B3LYP_6-31pgss_trans_H_B_eq_eq_01.log	9.1498	-1419.82288	0.00187114	16	4.91	0.137827914	0.018628271	1.86
s-smd-B3LYP_6-31pgss_trans_H_B_eq_eq_02.log	6.0812	-1419.823874	0.00087711	6	2.30	0.394964686	0.053381852	5.34
s-smd-B3LYP_6-31pgss_trans_H_B_eq_eq_21.log	4.6484	-1419.824173	0.00057791	3	1.52	0.542226102	0.073285117	7.33
s-smd-B3LYP_6-31pgss_trans_H_B_eq_eq_22.log	9.3986	-1419.822847	0.00190408	18	5.00	0.133102394	0.017989589	1.80
s-smd-B3LYP_6-31pgss_trans_H_B_eq_eq_24.log	7.6566	-1419.823211	0.00154066	15	4.05	0.195590207	0.026435192	2.64
-smd-B3LYP_6-31pgss_trans_H_B_eq_eq_611.log	6.8879	-1419.823607	0.0011442	12	3.00	0.297649843	0.040229166	4.02
-smd-B3LYP_6-31pgss_trans_H_B_eq_eq_612.log	4.9792	-1419.823324	0.00142731	14	3.75	0.220538479	0.029807101	2.98
-smd-B3LYP_6-31pgss_trans_H_B_eq_eq_613.log	9.1384	-1419.821132	0.00361941	26	9.50	0.021636675	0.002924327	0.29
-smd-B3LYP_6-31pgss_trans_H_B_eq_eq_621.log	8.6574	-1419.824751	0	1	0.00		0.135156011	13.52
-smd-B3LYP_6-31pgss_trans_H_B_eq_eq_622.log	6.9451	-1419.824136	0.00061576	4	1.62	0.520919561	0.07040541	7.04
s-smd-B3LYP_6-31pgss_trans_H_H_eq_eq_01.log	9.2185	-1419.82384	0.00091142	8	2.39	0.380870027	0.051476874	5.15
s-smd-B3LYP_6-31pgss_trans_H_H_eq_eq_02.log	6.3208	-1419.823801	0.00095055	9	2.50	0.365408196	0.049387114	4.94
s-smd-B3LYP_6-31pgss_trans_H_H_eq_eq_04.log	9.9075	-1419.8191	0.00565172	35	14.84	0.002514171	0.000339805	0.03
-smd-B3LYP_6-31pgss_trans_H_H_eq_eq_211.log	5.6644	-1419.818706	0.00604509	36	15.87	0.001657515	0.000224023	0.02
-smd-B3LYP_6-31pgss_trans_H_H_eq_eq_214.log	6.989	-1419.819345	0.00540623	33	14.19	0.00326071	0.000440705	0.04
s-smd-B3LYP_6-31pgss_trans_H_H_eq_eq_21.log	4.8307	-1419.823892	0.00085894	5	2.26	0.402639028	0.054419085	5.44
-smd-B3LYP_6-31pgss_trans_H_H_eq_eq_221.log	8.481	-1419.824548	0.00020361	2	0.53	0.806020525	0.108938519	10.89
-smd-B3LYP_6-31pgss_trans_H_H_eq_eq_224.log	7.2198	-1419.8237	0.00105084	11	2.76	0.328585341	0.044410284	4.44
s-smd-B3LYP_6-31pgss_trans_H_H_eq_eq_22.log	8.2316	-1419.822614	0.00213726	19	5.61	0.103975434	0.014052905	1.41
-smd-B3LYP_6-31pgss_trans_H_H_eq_eq_231.log	7.4572	-1419.823529	0.00122198	13	3.21	0.274112864	0.037048001	3.70
-smd-B3LYP_6-31pgss_trans_H_H_eq_eq_233.log	10.3938	-1419.820801	0.00394999	29	10.37	0.015245252	0.002060487	0.21
-smd-B3LYP_6-31pgss_trans_H_H_eq_eq_234.log	5.615	-1419.823726	0.00102573	10	2.69	0.337441085	0.045607191	4.56
s-smd-B3LYP_6-31pgss_trans_H_H_eq_eq_24.log	7.5085	-1419.82287	0.00188165	17	4.94	0.13630222	0.018422064	1.84
s-smd-B3LYP_6-31pgss_trans_H_H_eq_eq_31.log	9.3923	-1419.819377	0.00537384	32	14.11	0.003374508	0.000456085	0.05
s-smd-B3LYP_6-31pgss_trans_H_H_eq_eq_34.log	5.5518	-1419.81916	0.00559149	34	14.68	0.002679777	0.000362188	0.04
s-smd-B3LYP_6-31pgss_trans_H_H_eq_eq_61.log	4.7615	-1419.823865	0.00088654	7	2.33	0.391039634	0.052851357	5.29
							1.00	100.00

Supporting information

Table S2. Boltzmann weighted populations of conformations 1-36 used for DP4+ analysis of isomer 4*R*,10*R*,11*S*.

File name	Dipole	Energy	Ranking delta energy in a.u.	Rank	Ranking delta energy in kJ/mol	Ni/N0	Ni	Boltzmann population in %
s-smd-B3LYP_6-31pgss_cis_B_B_ax-C2_eq-C3_01.log	9.2155	-1419.81988035	0.004333	42	11.38	0.010158	0.001183	0.12
s-smd-B3LYP_6-31pgss_cis_B_B_ax-C2_eq-C3_02.log	6.1630	-1419.82091728	0.003296	33	8.65	0.030464	0.003548	0.35
s-smd-B3LYP_6-31pgss_cis_B_B_ax-C2_eq-C3_21.log	5.0164	-1419.82109801	0.003116	32	8.18	0.036890	0.004296	0.43
s-smd-B3LYP_6-31pgss_cis_B_B_ax-C2_eq-C3_22.log	10.3037	-1419.81990961	0.004304	41	11.30	0.010478	0.001220	0.12
s-smd-B3LYP_6-31pgss_cis_B_B_ax-C2_eq-C3_24.log	7.9715	-1419.82023447	0.003979	39	10.45	0.014781	0.001721	0.17
s-smd-B3LYP_6-31pgss_cis_B_B_ax-C2_eq-C3_41.log	9.3072	-1419.82126804	0.002946	31	7.73	0.044169	0.005144	0.51
s-smd-B3LYP_6-31pgss_cis_B_B_ax-C2_eq-C3_611.log	5.0467	-1419.81938409	0.004830	43	12.68	0.006006	0.000699	0.07
s-smd-B3LYP_6-31pgss_cis_B_B_ax-C2_eq-C3_612.log	6.8532	-1419.82070448	0.003509	36	9.21	0.024317	0.002832	0.28
s-smd-B3LYP_6-31pgss_cis_B_B_ax-C2_eq-C3_622.log	8.6983	-1419.82071448	0.003499	35	9.19	0.024575	0.002862	0.29
s-smd-B3LYP_6-31pgss_cis_B_H_ax-C2_eq-C3_01.log	9.0667	-1419.82170438	0.002509	28	6.59	0.070117	0.008166	0.82
s-smd-B3LYP_6-31pgss_cis_B_H_ax-C2_eq-C3_02.log	6.0370	-1419.82177099	0.002443	24	6.41	0.075242	0.008763	0.88
s-smd-B3LYP_6-31pgss_cis_B_H_ax-C2_eq-C3_21.log	4.6088	-1419.82176055	0.002453	25	6.44	0.074415	0.008666	0.87
s-smd-B3LYP_6-31pgss_cis_B_H_ax-C2_eq-C3_22.log	8.9694	-1419.82049512	0.003719	38	9.76	0.019481	0.002269	0.23
s-smd-B3LYP_6-31pgss_cis_B_H_ax-C2_eq-C3_24.log	7.4382	-1419.82076697	0.003447	34	9.05	0.025980	0.003026	0.30
s-smd-B3LYP_6-31pgss_cis_B_H_ax-C2_eq-C3_614.log	6.7827	-1419.81727846	0.006935	48	18.21	0.000646	0.000075	0.01
s-smd-B3LYP_6-31pgss_cis_B_H_ax-C2_eq-C3_621.log	8.4448	-1419.82243079	0.001783	17	4.68	0.151338	0.017625	1.76
s-smd-B3LYP_6-31pgss_cis_B_H_ax-C2_eq-C3_622.log	6.8968	-1419.82165058	0.002563	29	6.73	0.066233	0.007713	0.77
s-smd-B3LYP_6-31pgss_cis_B_H_ax-C2_eq-C3_631.log	7.1259	-1419.82145379	0.002760	30	7.25	0.053772	0.006262	0.63
s-smd-B3LYP_6-31pgss_cis_B_H_ax-C2_eq-C3_632.log	4.9571	-1419.82171080	0.002503	27	6.57	0.070595	0.008221	0.82
s-smd-B3LYP_6-31pgss_cis_H_B_eq-C2_ax-C3_01.log	9.2813	-1419.82227958	0.001934	20	5.08	0.128942	0.015016	1.50
s-smd-B3LYP_6-31pgss_cis_H_B_eq-C2_ax-C3_02.log	6.1491	-1419.82336130	0.000852	8	2.24	0.405463	0.047220	4.72
s-smd-B3LYP_6-31pgss_cis_H_B_eq-C2_ax-C3_21.log	4.7877	-1419.82357477	0.000639	3	1.68	0.508324	0.059199	5.92
s-smd-B3LYP_6-31pgss_cis_H_B_eq-C2_ax-C3_22.log	9.5155	-1419.82220039	0.002013	22	5.29	0.118569	0.013808	1.38
s-smd-B3LYP_6-31pgss_cis_H_B_eq-C2_ax-C3_23.log	7.8486	-1419.82272581	0.001488	16	3.91	0.206846	0.024089	2.41
s-smd-B3LYP_6-31pgss_cis_H_B_eq-C2_ax-C3_41.log	9.2934	-1419.82350777	0.000706	5	1.85	0.473503	0.055144	5.51
s-smd-B3LYP_6-31pgss_cis_H_B_eq-C2_ax-C3_611.log	7.0250	-1419.82301647	0.001197	14	3.14	0.281411	0.032773	3.28
s-smd-B3LYP_6-31pgss_cis_H_B_eq-C2_ax-C3_612.log	5.0499	-1419.82313550	0.001078	12	2.83	0.319221	0.037176	3.72
s-smd-B3LYP_6-31pgss_cis_H_B_eq-C2_ax-C3_613.log	9.0997	-1419.82056778	0.003646	37	9.57	0.021039	0.002450	0.25
s-smd-B3LYP_6-31pgss_cis_H_B_eq-C2_ax-C3_621.log	8.8017	-1419.82421364	0.000000	1	0.00		0.116459	11.65
s-smd-B3LYP_6-31pgss_cis_H_B_eq-C2_ax-C3_622.log	6.9926	-1419.82357323	0.000640	4	1.68	0.507496	0.059102	5.91
s-smd-B3LYP_6-31pgss_cis_H_H_eq-C2_ax-C3_01.log	9.3305	-1419.82323241	0.000981	10	2.58	0.353726	0.041194	4.12
s-smd-B3LYP_6-31pgss_cis_H_H_eq-C2_ax-C3_02.log	6.3513	-1419.82327666	0.000937	9	2.46	0.370698	0.043171	4.32
s-smd-B3LYP_6-31pgss_cis_H_H_eq-C2_ax-C3_04.log	9.9627	-1419.81857737	0.005636	46	14.80	0.002556	0.000298	0.03
s-smd-B3LYP_6-31pgss_cis_H_H_eq-C2_ax-C3_211.log	5.7620	-1419.81816032	0.006053	47	15.89	0.001643	0.000191	0.02
s-smd-B3LYP_6-31pgss_cis_H_H_eq-C2_ax-C3_214.log	7.0031	-1419.81884670	0.005367	45	14.09	0.003399	0.000396	0.04
s-smd-B3LYP_6-31pgss_cis_H_H_eq-C2_ax-C3_21.log	4.8343	-1419.82338975	0.000824	7	2.16	0.417867	0.048664	4.87
s-smd-B3LYP_6-31pgss_cis_H_H_eq-C2_ax-C3_221.log	8.6024	-1419.82390113	0.000313	2	0.82	0.718217	0.083643	8.36
s-smd-B3LYP_6-31pgss_cis_H_H_eq-C2_ax-C3_224.log	7.2319	-1419.82310449	0.001109	13	2.91	0.308907	0.035975	3.60
s-smd-B3LYP_6-31pgss_cis_H_H_eq-C2_ax-C3_22.log	8.3090	-1419.82205173	0.002162	23	5.68	0.101296	0.011797	1.18
s-smd-B3LYP_6-31pgss_cis_H_H_eq-C2_ax-C3_231.log	7.5658	-1419.82295543	0.001258	15	3.30	0.263794	0.030721	3.07
s-smd-B3LYP_6-31pgss_cis_H_H_eq-C2_ax-C3_233.log	10.3549	-1419.82018250	0.004031	40	10.58	0.013990	0.001629	0.16
s-smd-B3LYP_6-31pgss_cis_H_H_eq-C2_ax-C3_234.log	5.6669	-1419.82317310	0.001041	11	2.73	0.332189	0.038686	3.87
s-smd-B3LYP_6-31pgss_cis_H_H_eq-C2_ax-C3_24.log	7.4820	-1419.82222888	0.001985	21	5.21	0.122201	0.014231	1.42
s-smd-B3LYP_6-31pgss_cis_H_H_eq-C2_ax-C3_31.log	9.5039	-1419.81889940	0.005314	44	13.95	0.003594	0.000419	0.04

s-smd-B3LYP_6-31pgss_cis_H_H_eq-C2_ax-C3_64.log	7.3101	-1419.82228656	0.001927	19	5.06	0.129899	0.015128	1.51
s-smd-B3LYP_6-31pgss_ciss_B_H_ax-C2_eq-C3_21.log	4.5836	-1419.82171832	0.002495	26	6.55	0.071160	0.008287	0.83
s-smd-B3LYP_6-31pgss_ciss_H_H_eq-C2_ax-C3_21.log	4.8845	-1419.82347708	0.000737	6	1.93	0.458360	0.053380	5.34
s-smd-B3LYP_6-31pgss_ciss_H_H_eq-C2_ax-C3_24.log	7.6565	-1419.82230725	0.001906	18	5.01	0.132777	0.015463	1.55
							1	100

Supporting information

Table S3. Boltzmann weighted populations of conformations 1-36 used for DP4+ analysis of isomer 4*S*,10*R*,11*R*.

File name	Dipole	Energy	Ranking delta energy in a.u.	Rank	Ranking delta energy in kJ/mol	Ni/N0	Ni	Boltzmann population in %
s-smd-B3LYP_6-31pgss_trans_B_B_eq_eq_01.log	9.2736	-1419.822436	0.00251354	16	6.59929927	0.069799824	0.012420696	1.24
s-smd-B3LYP_6-31pgss_trans_B_B_eq_eq_02.log	6.1839	-1419.823517	0.0014324	10	3.7607662	0.21935278	0.039033255	3.90
s-smd-B3LYP_6-31pgss_trans_B_B_eq_eq_21.log	5.0482	-1419.823599	0.00135042	9	3.54552771	0.239249613	0.042573844	4.26
s-smd-B3LYP_6-31pgss_trans_B_B_eq_eq_22.log	10.2712	-1419.822355	0.00259434	17	6.81143967	0.064075055	0.011401989	1.14
s-smd-B3LYP_6-31pgss_trans_B_B_eq_eq_24.log	8.0456	-1419.822755	0.00219448	15	5.76160724	0.0978614	0.017414181	1.74
s-smd-B3LYP_6-31pgss_trans_B_B_eq_eq_41.log	9.3611	-1419.82378	0.00116956	8	3.07067978	0.289761641	0.051562328	5.16
s-smd-B3LYP_6-31pgss_trans_B_B_eq_eq_611.log	5.0855	-1419.821782	0.00316735	24	8.315877425	0.034923941	0.006214624	0.62
s-smd-B3LYP_6-31pgss_trans_B_B_eq_eq_612.log	6.9301	-1419.823169	0.00178054	13	4.67480777	0.1517086	0.02699615	2.70
s-smd-B3LYP_6-31pgss_trans_B_B_eq_eq_613.log	8.2605	-1419.820494	0.00445526	32	11.69728513	0.008927442	0.001588615	0.16
s-smd-B3LYP_6-31pgss_trans_B_B_eq_eq_622.log	8.763	-1419.823193	0.00175649	12	4.611664495	0.155622508	0.027692619	2.77
s-smd-B3LYP_6-31pgss_trans_B_H_eq_eq_01.log	9.1176	-1419.824199	0.00075044	3	1.97028022	0.451671095	0.080373693	8.04
s-smd-B3LYP_6-31pgss_trans_B_H_eq_eq_02.log	6.0666	-1419.824192	0.00075765	4	1.989210076	0.448235176	0.07976228	7.98
s-smd-B3LYP_6-31pgss_trans_B_H_eq_eq_04.log	10.3897	-1419.819408	0.00554126	36	14.54857813	0.002826199	0.000502915	0.05
s-smd-B3LYP_6-31pgss_trans_B_H_eq_eq_21.log	4.6411	-1419.82427	0.00067985	2	1.784946175	0.486733654	0.086612984	8.66
s-smd-B3LYP_6-31pgss_trans_B_H_eq_eq_22.log	8.9378	-1419.823041	0.00190868	14	5.01123934	0.132455507	0.023570112	2.36
s-smd-B3LYP_6-31pgss_trans_B_H_eq_eq_24.log	7.5105	-1419.823309	0.00164009	11	4.306056295	0.176040494	0.031325947	3.13
s-smd-B3LYP_6-31pgss_trans_B_H_eq_eq_31.log	9.2494	-1419.819853	0.00509657	33	13.38104454	0.004526323	0.000805447	0.08
s-smd-B3LYP_6-31pgss_trans_B_H_eq_eq_34.log	5.5466	-1419.819628	0.00532159	35	13.97183455	0.003566512	0.000634652	0.06
s-smd-B3LYP_6-31pgss_trans_B_H_eq_eq_611.log	5.224	-1419.819083	0.00586605	37	15.40131428	0.002003596	0.000356535	0.04
s-smd-B3LYP_6-31pgss_trans_B_H_eq_eq_614.log	6.8111	-1419.819776	0.00517362	34	13.58333931	0.004171623	0.000742329	0.07
s-smd-B3LYP_6-31pgss_trans_B_H_eq_eq_621.log	8.494	-1419.824949	0	1	0		0.177947391	17.79
s-smd-B3LYP_6-31pgss_trans_B_H_eq_eq_622.log	6.9238	-1419.824096	0.00085348	6	2.24081174	0.404974138	0.072064091	7.21
s-smd-B3LYP_6-31pgss_trans_B_H_eq_eq_631.log	7.2341	-1419.824	0.00094919	7	2.492098345	0.365934907	0.065117162	6.51
s-smd-B3LYP_6-31pgss_trans_B_H_eq_eq_632.log	5.024	-1419.824156	0.00079349	5	2.083307996	0.43153966	0.076791356	7.68
s-smd-B3LYP_6-31pgss_trans_B_H_eq_eq_634.log	9.3997	-1419.820845	0.00410473	30	10.77696862	0.012940745	0.002302772	0.23
s-smd-B3LYP_6-31pgss_trans_H_B_ax_ax_01.log	9.2188	-1419.8209	0.0040491	28	10.63091205	0.013726103	0.002442524	0.24
s-smd-B3LYP_6-31pgss_trans_H_B_ax_ax_02.log	6.1106	-1419.822001	0.00294865	20	7.741680575	0.044026894	0.007834471	0.78
s-smd-B3LYP_6-31pgss_trans_H_B_ax_ax_21.log	4.7078	-1419.822258	0.00269138	18	7.066218191	0.057816764	0.010288342	1.03
s-smd-B3LYP_6-31pgss_trans_H_B_ax_ax_22.log	9.3935	-1419.820858	0.00409139	29	10.74194445	0.013124877	0.002335538	0.23
s-smd-B3LYP_6-31pgss_trans_H_B_ax_ax_23.log	7.6918	-1419.82123	0.00371968	26	9.76601984	0.01945672	0.003462272	0.35
s-smd-B3LYP_6-31pgss_trans_H_B_ax_ax_41.log	9.2192	-1419.822064	0.00288539	19	7.575591445	0.047077735	0.00837736	0.84
s-smd-B3LYP_6-31pgss_trans_H_H_ax_ax_01.log	9.2747	-1419.821894	0.00305559	22	8.022451545	0.039312364	0.006995533	0.70
s-smd-B3LYP_6-31pgss_trans_H_H_ax_ax_02.log	6.3328	-1419.821885	0.00306449	23	8.045818495	0.038943543	0.006929902	0.69
s-smd-B3LYP_6-31pgss_trans_H_H_ax_ax_21.log	4.7877	-1419.821963	0.00298673	21	7.841659616	0.042286574	0.007524785	0.75
s-smd-B3LYP_6-31pgss_trans_H_H_ax_ax_22.log	8.2097	-1419.820723	0.00422616	31	11.09578308	0.011379037	0.00202487	0.20
s-smd-B3LYP_6-31pgss_trans_H_H_ax_ax_24.log	7.3548	-1419.820919	0.00403069	27	10.5825766	0.013996365	0.002490617	0.25
s-smd-B3LYP_6-31pgss_trans_H_H_ax_ax_64.log	6.8026	-1419.821236	0.00371328	25	9.74921664	0.019589052	0.003485821	0.35
							1.00	100.00

Table S4. Boltzmann weighted populations of conformations 1-36 used for DP4⁺ analysis of isomer 4*S*,10*R*,11*S*.

File name	Dipole	Energy	Ranking delta energy in a.u.	Rank	Ranking delta energy in kJ/mol	Ni/N0	Ni	Boltzmann population in %
s-smd-B3LYP_6-31pgss_cis_B_B_eq_ax_0001.log	9.3828	-1419.82183597	0.002440	18	6.41	0.075421	0.012341	1.23
s-smd-B3LYP_6-31pgss_cis_B_B_eq_ax_0002.log	6.2324	-1419.82286886	0.001408	10	3.70	0.225210	0.036850	3.68
s-smd-B3LYP_6-31pgss_cis_B_B_eq_ax_0021.log	5.1138	-1419.82309787	0.001179	7	3.09	0.287028	0.046965	4.70
s-smd-B3LYP_6-31pgss_cis_B_B_eq_ax_0022.log	10.2202	-1419.82184790	0.002428	17	6.38	0.076380	0.012498	1.25
s-smd-B3LYP_6-31pgss_cis_B_B_eq_ax_0024.log	8.1352	-1419.82207176	0.002205	15	5.79	0.096816	0.015841	1.58
s-smd-B3LYP_6-31pgss_cis_B_B_eq_ax_0601.log	8.8696	-1419.82266529	0.001611	12	4.23	0.181531	0.029703	2.97
s-smd-B3LYP_6-31pgss_cis_B_B_eq_ax_0602.log	7.0430	-1419.82305243	0.001224	8	3.21	0.273542	0.044758	4.48
s-smd-B3LYP_6-31pgss_cis_B_B_eq_ax_0632.log	4.9452	-1419.82300621	0.001270	9	3.33	0.260474	0.042620	4.26
s-smd-B3LYP_6-31pgss_cis_B_H_eq_ax_0001.log	9.2362	-1419.82366422	0.000612	4	1.61	0.522910	0.085561	8.56
s-smd-B3LYP_6-31pgss_cis_B_H_eq_ax_0002.log	6.1441	-1419.82366976	0.000607	3	1.59	0.525987	0.086064	8.61
s-smd-B3LYP_6-31pgss_cis_B_H_eq_ax_0021.log	4.7073	-1419.82374745	0.000529	2	1.39	0.571097	0.093445	9.34
s-smd-B3LYP_6-31pgss_cis_B_H_eq_ax_0022.log	8.8838	-1419.82252331	0.001753	13	4.60	0.156187	0.025556	2.56
s-smd-B3LYP_6-31pgss_cis_B_H_eq_ax_0024.log	7.6530	-1419.82270609	0.001570	11	4.12	0.189548	0.031015	3.10
s-smd-B3LYP_6-31pgss_cis_B_H_eq_ax_0611.log	6.8702	-1419.81929315	0.004983	37	13.08	0.005104	0.000835	0.08
s-smd-B3LYP_6-31pgss_cis_B_H_eq_ax_0612.log	5.4154	-1419.81859324	0.005683	38	14.92	0.002432	0.000398	0.04
s-smd-B3LYP_6-31pgss_cis_B_H_eq_ax_0621.log	6.9911	-1419.82355311	0.000723	5	1.90	0.464857	0.076062	7.61
s-smd-B3LYP_6-31pgss_cis_B_H_eq_ax_0622.log	8.6208	-1419.82427638	0.000000	1	0.00		0.163625	16.36
s-smd-B3LYP_6-31pgss_cis_B_H_eq_ax_0631.log	7.4063	-1419.82346389	0.000812	6	2.13	0.422943	0.069204	6.92
s-smd-B3LYP_6-31pgss_cis_B_H_eq_ax_0635.log	9.5576	-1419.82028637	0.003990	34	10.48	0.014613	0.002391	0.24
s-smd-B3LYP_6-31pgss_cis_H_B_ax_eq_0001.log	9.0980	-1419.82039786	0.003879	32	10.18	0.016444	0.002691	0.27
s-smd-B3LYP_6-31pgss_cis_H_B_ax_eq_0002.log	6.0610	-1419.82140919	0.002867	21	7.53	0.047994	0.007853	0.79
s-smd-B3LYP_6-31pgss_cis_H_B_ax_eq_0021.log	4.5863	-1419.82167789	0.002598	19	6.82	0.063794	0.010438	1.04
s-smd-B3LYP_6-31pgss_cis_H_B_ax_eq_0022.log	9.3283	-1419.82038361	0.003893	33	10.22	0.016198	0.002650	0.27
s-smd-B3LYP_6-31pgss_cis_H_B_ax_eq_0024.log	7.5056	-1419.82062290	0.003653	31	9.59	0.020870	0.003415	0.34
s-smd-B3LYP_6-31pgss_cis_H_B_ax_eq_0611.log	6.8287	-1419.82110914	0.003167	27	8.32	0.034928	0.005715	0.57
s-smd-B3LYP_6-31pgss_cis_H_B_ax_eq_0612.log	5.1500	-1419.81974259	0.004534	36	11.90	0.008215	0.001344	0.13
s-smd-B3LYP_6-31pgss_cis_H_B_ax_eq_0621.log	8.6064	-1419.82209984	0.002177	14	5.71	0.099739	0.016320	1.63
s-smd-B3LYP_6-31pgss_cis_H_B_ax_eq_0622.log	6.9282	-1419.82144533	0.002831	20	7.43	0.049867	0.008159	0.82
s-smd-B3LYP_6-31pgss_cis_H_H_ax_eq_0001.log	9.1552	-1419.82128079	0.002996	24	7.86	0.041892	0.006854	0.69
s-smd-B3LYP_6-31pgss_cis_H_H_ax_eq_0002.log	6.3035	-1419.82120872	0.003068	26	8.05	0.038813	0.006351	0.64
s-smd-B3LYP_6-31pgss_cis_H_H_ax_eq_0021.log	4.7112	-1419.82140244	0.002874	22	7.55	0.047652	0.007797	0.78
s-smd-B3LYP_6-31pgss_cis_H_H_ax_eq_0022.log	8.1402	-1419.82011403	0.004162	35	10.93	0.012175	0.001992	0.20
s-smd-B3LYP_6-31pgss_cis_H_H_ax_eq_0024.log	6.6639	-1419.82072709	0.003549	30	9.32	0.023305	0.003813	0.38
s-smd-B3LYP_6-31pgss_cis_H_H_ax_eq_0061.log	4.7111	-1419.82140227	0.002874	23	7.55	0.047644	0.007796	0.78
s-smd-B3LYP_6-31pgss_cis_H_H_ax_eq_0214.log	6.9669	-1419.81693272	0.007344	39	19.28	0.000419	0.000069	0.01
s-smd-B3LYP_6-31pgss_cis_H_H_ax_eq_0221.log	8.4264	-1419.82191636	0.002360	16	6.20	0.082124	0.013437	1.34
s-smd-B3LYP_6-31pgss_cis_H_H_ax_eq_0224.log	7.2062	-1419.82108949	0.003187	28	8.37	0.034209	0.005597	0.56
s-smd-B3LYP_6-31pgss_cis_H_H_ax_eq_0231.log	7.3768	-1419.82107177	0.003205	29	8.41	0.033573	0.005493	0.55
s-smd-B3LYP_6-31pgss_cis_H_H_ax_eq_0234.log	5.5753	-1419.82122823	0.003048	25	8.00	0.039623	0.006483	0.65
							1	100

Table S5. Coefficients of determination R^2 of the linear regressions made between experimental chemical shifts and theorical chemical shifts of each isomer.

	(4*R*,10*R*,11*R*)	(4*S*,10*R*,11*R*)	(4*S*,10*R*,11*S*)	(4*R*,10*R*,11*S*)
δ^{1}H R^2	0.994	0.9888	0.9864	0.9862
δ^{13}C R^2	0.9994	0.9994	0.9991	0.995

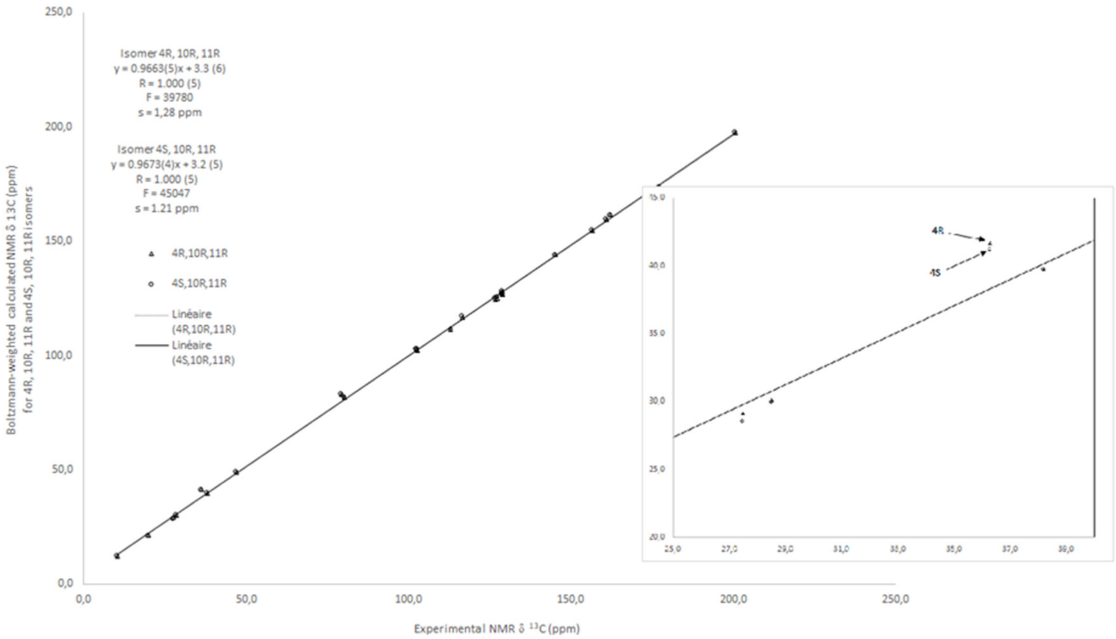

Figure S7. Plot of Boltzmann-weighted calculated NMR *δ* ^{13}C of 4*R*,10*R*,11*R* and 4*S*,10*R*,11*R* isomers versus experimental NMR *δ* ^{13}C of isocaloteysmanic acid (1). Statistics for the regression of calculated versus experimental chemical shifts for both isomers. The slope, the intercept, the correlation coefficient (r) are followed by their standard error on the last digit(s) in brackets. F is the Fisher F-statistic and s the standard error of the fit.

Table S6. Enthalpies and Boltzmann populations of conformations A_1-A_{14} of (4*R*,10*R*,11*R*) and conformations B_1-B_{14} of (4S,10*R*,11*R*), calculated using GD3BJ-B3LYP/6-311G(d,p) level.

**(4*R*,10*R*,11*R*)**				**(4*S*,10*R*,11*R*)**			
Conformations	H^{298K} (in a.u)	H^{298K} (in kcal.mol^{-1})	Boltzmann Distribution	Conformations	H^{298K} (in a.u)	H^{298K} (in kcal.mol^{-1})	Boltzmann Distribution
**A_1**	-1419.710264	0.00	**0.49**	**B_1**	-1419.711926	0.00	**0.26**
**A_2**	-1419.708957	0.82	**0.12**	**B_2**	-1419.711858	0.04	**0.24**
**A_3**	-1419.708865	0.88	**0.11**	**B_3**	-1419.711694	0.15	**0.20**
**A_4**	-1419.708753	0.95	**0.10**	**B_4**	-1419.711092	0.52	**0.11**
**A_5**	-1419.708256	1.26	**0.06**	**B_5**	-1419.710811	0.70	**0.08**
**A_6**	-1419.708235	1.27	**0.06**	**B_6**	-1419.710712	0.76	**0.07**
A_7	-1419.70766	1.63	0.03	B_7	-1419.708998	1.84	0.01
A_8	-1419.706859	2.14	0.01	B_8	-1419.708554	2.12	0.01
A_9	-1419.706849	2.14	0.01	B_9	-1419.70806	2.43	0.00
A_{10}	-1419.706536	2.34	0.01	B_{10}	-1419.708046	2.43	0.00
A_{11}	-1419.690066	12.67	0.00	B_{11}	-1419.707445	2.81	0.00
A_{12}	-1419.689725	12.89	0.00	B_{12}	-1419.707248	2.93	0.00
A_{13}	-1419.688123	13.89	0.00	B_{13}	-1419.707037	3.07	0.00
A_{14}	-1419.687138	14.51	0.00	B_{14}	-1419.706552	3.37	0.00

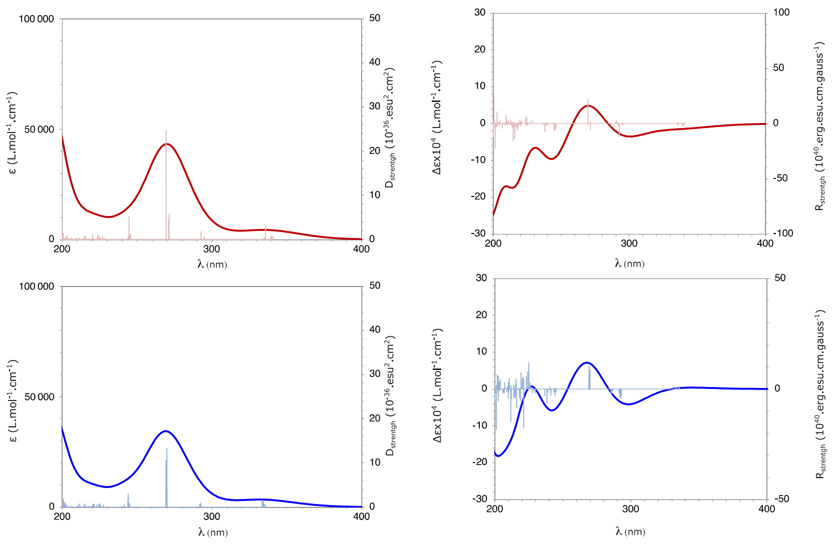

Figure S8. UV (left) and ECD (right) spectra calculated using SMD(CH_3OH)/CAM-B3LYP/6-31++G(d,p)//GD3BJ-B3LYP/6-311G(d,p) level for (4*R*,10*R*,11*R*) (red) and (4*S*,10*R*,11*R*) (blue).

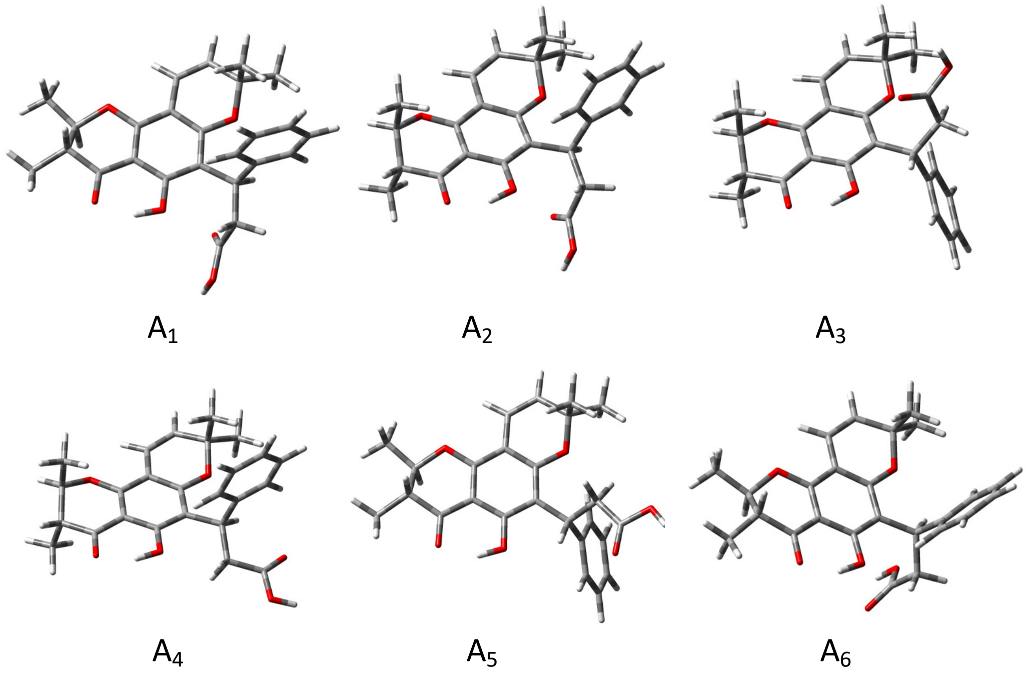

Figure S9. Conformations A1 to A6 selected to build the UV and ECD spectra of the diastereomer (4*R*,10*R*,11*R*). Geometries optimized to the level GD3BJ-B3LYP/6-311G(d,p).

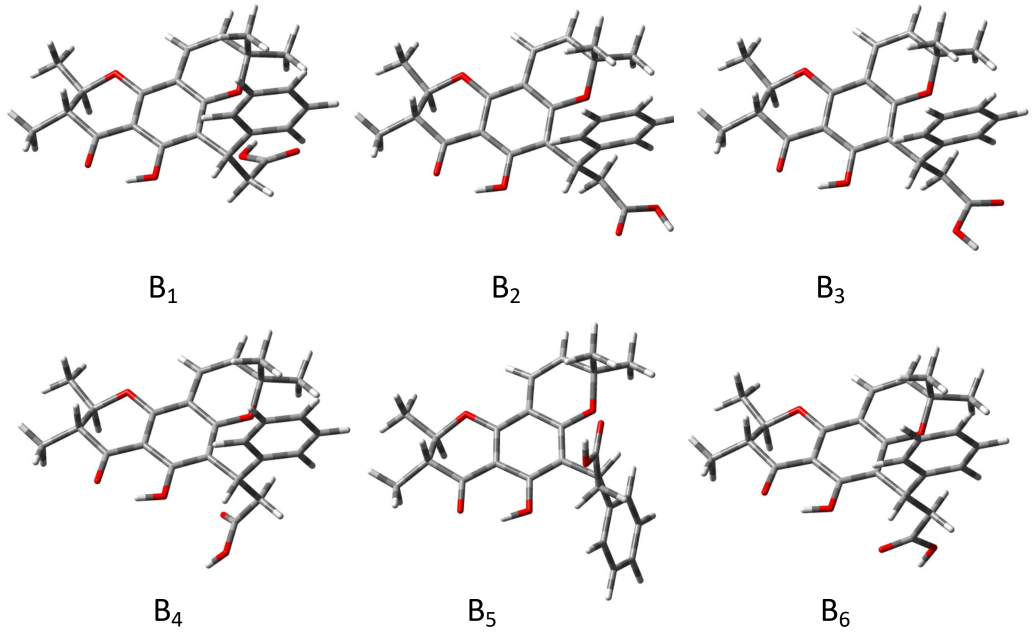

Figure S10. Conformations B1 to B6 selected to build the UV and ECD spectra of the diastereomer (4*S*,10*R*,11*R*). Geometries optimized to the level GD3BJ-B3LYP/6-311G(d,p).

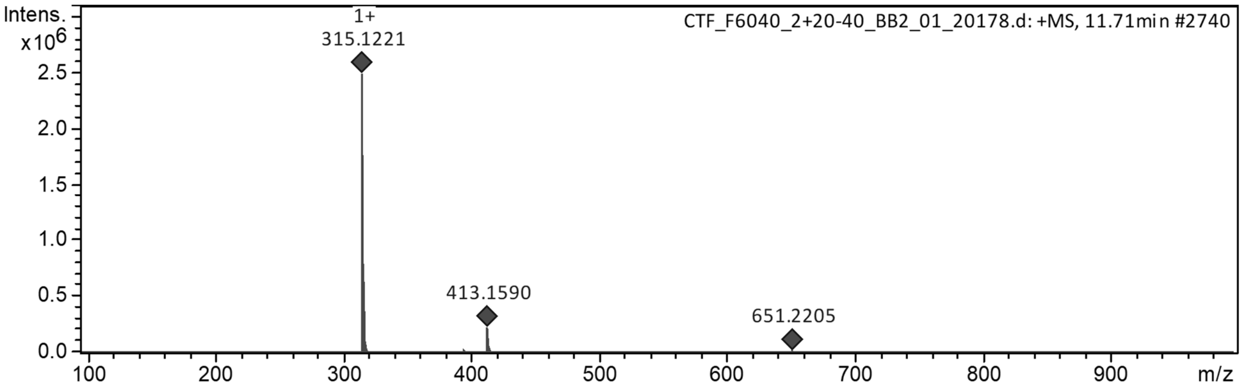

Figure S11. HRESIMS spectrum for 6-(4-hydroxy-3-methylbutyl)-1,5-dihydroxyxanthone (3).

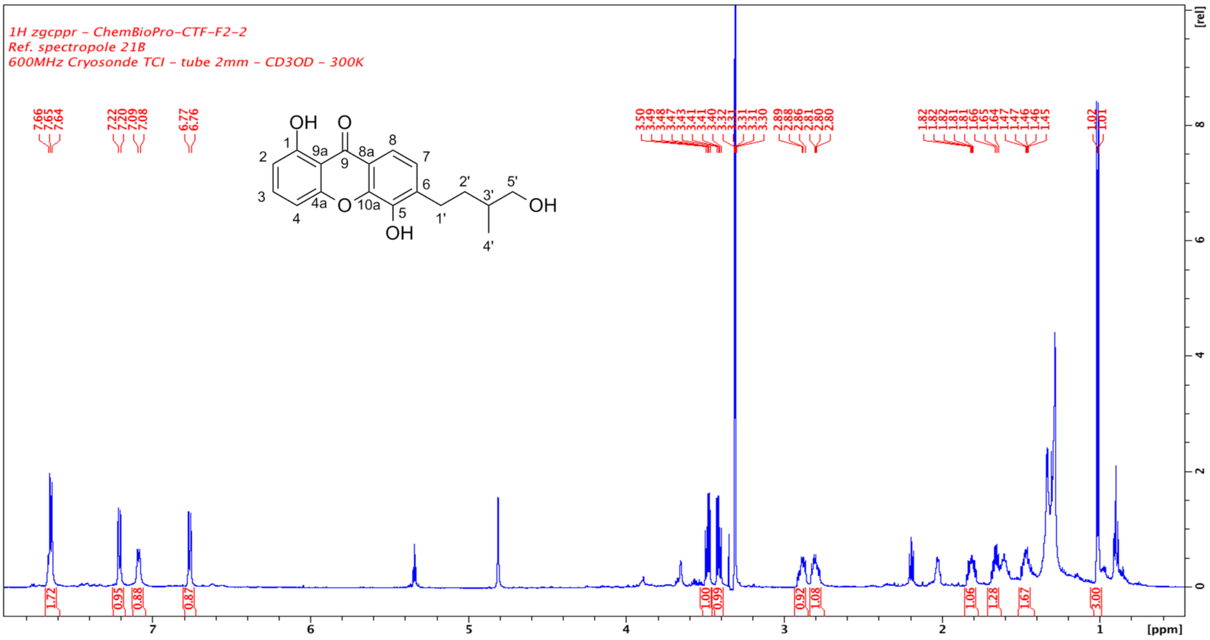

Figure S12. ^{1}H NMR (600 MHz, CD_3OD) spectrum for 6-(4-hydroxy-3-methylbutyl)-1,5-dihydroxyxanthone (3).

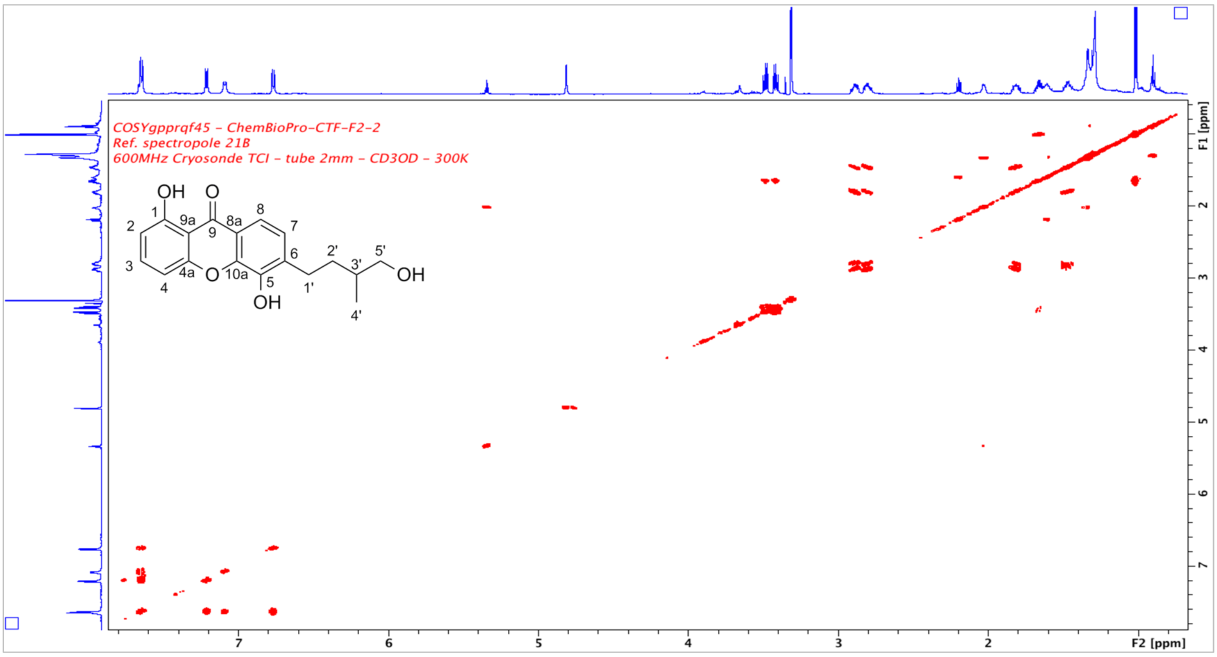

Figure S13. ^{1}H-^{1}H COSY NMR (600 MHz, CD_3OD) spectrum for 6-(4-hydroxy-3-methylbutyl)-1,5-dihydroxyxanthone (3).

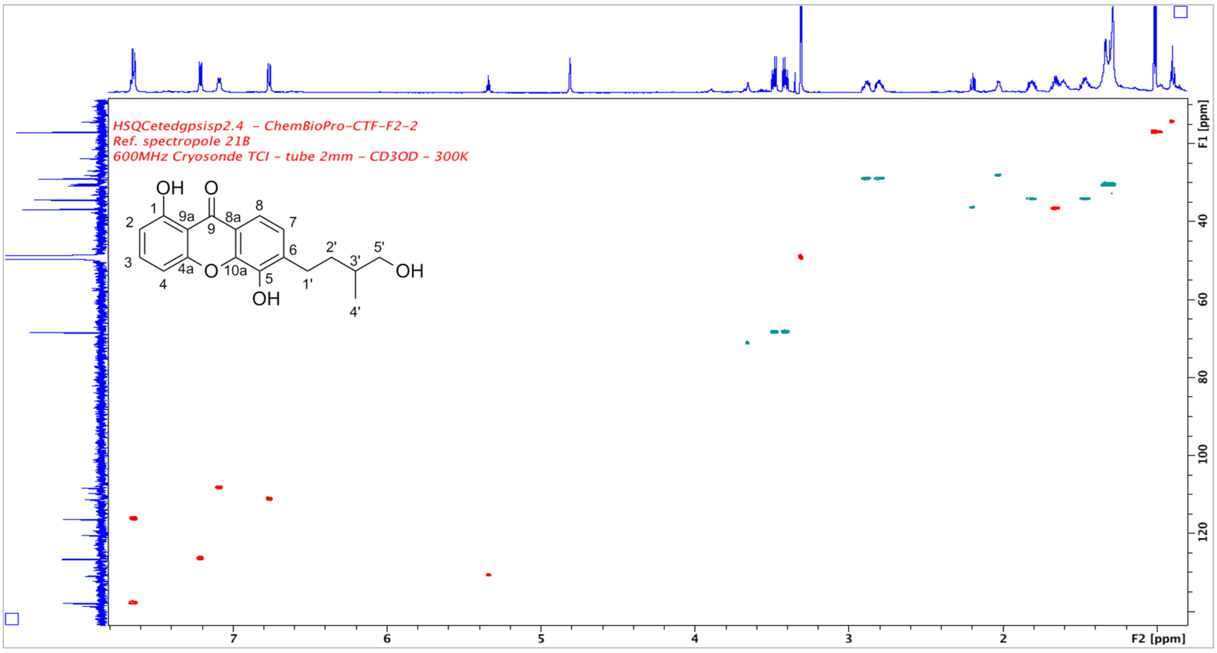

Figure S14. ^{1}H-^{13}C HSQC NMR (600 MHz, CD_3OD) spectrum for 6-(4-hydroxy-3-methylbutyl)-1,5-dihydroxyxanthone (3).

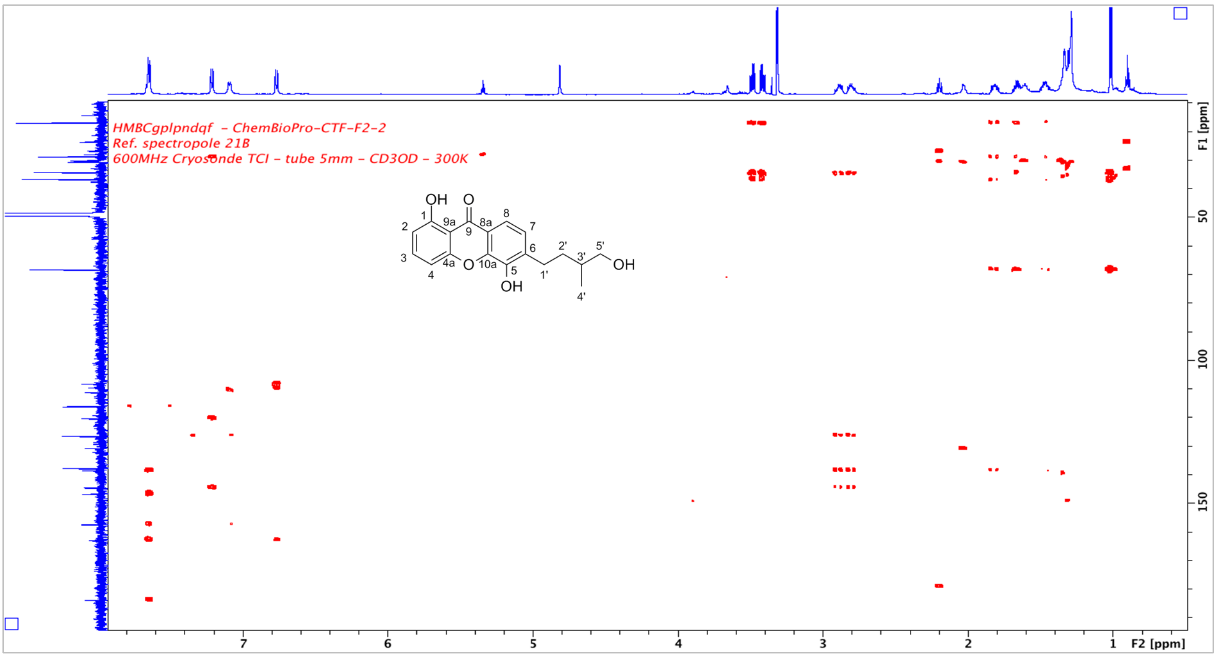

Figure S15. ^{1}H-^{13}C HMBC NMR (600 MHz, CD_3OD) spectrum for 6-(4-hydroxy-3-methylbutyl)-1,5-dihydroxyxanthone (3).
